# Editorial: Leukocyte biology and muscle pathology: Leukocytes as potential targets to control muscle inflammation, mass, and pain

**DOI:** 10.3389/fphar.2023.1154559

**Published:** 2023-02-09

**Authors:** Miriam S. N. Hohmann, Waldiceu A. Verri, Flavia A. Guarnier, Barbara St. Pierre Schneider, Sergio M. Borghi

**Affiliations:** ^1^ Department of Medicine, Cedars-Sinai Medical Center, Women’s Guild Lung Institute, Los Angeles, CA, United States; ^2^ Department of General Pathology, Londrina State University, Londrina, Paraná, Brazil; ^3^ College of Nursing and Health Innovation, University of Texas at Arlington, Arlington, TX, United States; ^4^ Center for Research in Health Sciences, University of Northern Londrina, Londrina, Paraná, Brazil

**Keywords:** leukocytes, skeletal muscle, spinal cord, inflammation, mass, pain, exercise, myopathology

Myositis reflects a term in which muscle is affected by inflammation, and the clinical manifestations include edema, weakness, and myalgia. Infections, autoimmunity, traumatic injuries (e.g., exercise and/or sports injuries), and medicine-related adverse effects represent the most common causes of myositis. Myositis as well as aging and reduced physical activity can induce atrophy or sarcopenia, which contributes to physical frailty and disabilities. Leukocytes are fundamental effector cells of immunity, which aims to protect the organism from intrinsic and extrinsic harmful agents. These cells also participate in the inflammatory response to traumatic injuries induced by exercise or other insults. However, with excessive activation, leukocytes may contribute to exaggerated inflammatory responses, pain, and cellular impairment through different pathways. Relevant to skeletal muscle, these pathways may modulate muscle cell damage and death, atrophy, and metabolic disorders. The scope of this Research Topic covers these different subjects in seven articles—one brief research report, three original articles, and three reviews.

In the first original article, Borghi et al. demonstrated in depth neuroinflammatory mechanisms triggered by a murine model of for delayed onset muscle soreness (DOMS) induced by acute swimming (in which male mice swam for two uninterrupted hours). DOMS is a common pain response following unaccustomed exercise-induced muscle damage. The participation of spinal cord glial cells in DOMS was demonstrated since intra-thecal treatments with the glial inhibitors, fluorocitrate, *α*-aminoadipate, and minocycline, reduced DOMS and nuclear factor κB (NFκB) activation in these spinal cord cells. These pharmacological approaches were accompanied by cellular and molecular biology results. The contribution of spinal cord glial was demonstrated to be dependent on regulating reactive oxygen species, C-X_3_-C motif chemokine ligand 1 (CX_3_CL1), tumor necrosis factor (TNF)-α, IL-1β, and IL-10 production. Thus, these data unveiled spinal cord mechanisms involved in DOMS.

In the brief research report, Borghi et al. studied the repurposing of pentoxifylline (an hemorheological drug prescribed for the treatment of peripheral vascular diseases) for DOMS taking as a background the mechanisms described previously (Borghi et al.). Pentoxifylline reduced DOMS induced by acute swimming (same protocol as described above). Specifically, there was a reduction in myalgia, neutrophil migration into the muscle, and oxidative stress/cytokine production in the muscle. Furthermore, the drug inhibited spinal astrocyte and microglial activation as well as cytokine production. Thus, demonstrating that pentoxifylline targets peripheral and spinal mechanisms to reduce DOMS.

In the second original article, Rodrigues et al. used male Wistar rats to evaluate the effects and mechanisms of action of resistance exercise training (RET; 3 times per week, for 14 days), whey protein supplementation (WPS; for 14 days), and the combination of both protocols (RET + WPS) upon pain induced by direct muscle trauma. All paradigms inhibited mechanical allodynia and leukocyte recruitment into the injured muscles. RET alone or RET + WPS improved gait performance and efficiently reduced muscle interleukin (IL)-6 levels. Regarding central events related to the muscle trauma, RET alone or RET + WPS inhibited spinal microglia activation; however, none of the approaches inhibited astrocyte activation. Therefore, RES and WPS can be effectively used as non-pharmacological approaches to promote analgesic effects after acute muscle trauma.

Finally, Bivona III et al. evaluated the influence of skeletal muscle-derived cytokines on circulating cytokine levels using a murine acute endotoxemia experimental model and specific muscle knockout conditional approaches. The researchers demonstrated that genetic deletion of muscle IL-6 and C-C motif chemokine ligand 2 (CCL2) led to a substantial reduction of these cytokines systemically at 3 h after lipopolysaccharide injection. Moreover, after toll-like receptor 4 (TLR4) muscle deletion, reductions in the expression of IL-6, CCL2, and C-X-C motif chemokine ligand 1 (CXCL1) in the gastrocnemius were observed. These data highlight the direct contribution of muscle fibers to the early systemic cytokine response in endotoxemic conditions.

Collectively, these preclinical data reinforce the fact that preclinical studies are, at this moment, fundamental to reveal the deep mechanisms underneath muscle pain related to inflammation.

As part of this Research Topic, we present one mini review and two reviews. In the mini review, Howard et al. discuss the contribution of mineralocorticoid receptor (MR) signaling in the skeletal muscle microenvironment of muscular dystrophy and acute injury pathologies. The study is to establish the differential role of MR signaling in the muscle and immune cells in the mentioned conditions, besides the repercussions of pharmacological targeting of these pathways in both. The potential advantages of MR antagonists’ therapy over and/or alternated with glucocorticoids agonist therapy are also discussed. Saito and Chikenji conducted a comprehensive review about the differential roles of senescent cells upon inflammation, tissue repair and regeneration, and aging and its related diseases, focusing on skeletal muscle tissue. The beneficial and detrimental effects of cellular senescence in the context of acute and chronic inflammatory states are addressed, as well as the application of the therapeutic target of senescence (senotherapy) to manage age-related disease, with the aim of improving muscle regeneration. To this end, Deng et al. reviewed the evolution of drug development in the treatment of Duchenne muscular dystrophy (DMD). DMD is a genetic, multi-systemic, progressive, and incurable disease that occurs after mutations in the dystrophin gene. Therapeutic strategies currently used for DMD are discussed in detail. Data regarding DMD clinical trials in development and information on approved drugs and new candidates for DMD treatment are addressed, providing a current overview of the advances and perspectives in DMD management.

In conclusion, the original reports and review articles address a variety of conditions that result in muscle inflammation, muscle pain, or systemic inflammation. Local, peripheral, and/or central mechanisms can regulate this inflammation and pain and involve leukocytes and other cells. Therefore, both muscle fibers and leukocytes may be targets of intervention to ameliorate muscle pain and reduce systemic and muscle inflammation.

